# Prevalence and factors associated with anxiety and depression among community-dwelling older adults in Hunan, China: a cross-sectional study

**DOI:** 10.1186/s12888-023-04583-5

**Published:** 2023-02-15

**Authors:** Lulu Lu, Hongxian Shen, Liwen Tan, Qiuping Huang, Qiongni Chen, Mining Liang, Li He, Yang Zhou

**Affiliations:** 1 Clinical Nursing Teaching and Research Section, The Second Xiangya Hospital, Central South University, Changsha, Hunan Province China; 2 Department of Psychiatry, The Second Xiangya Hospital, Central South University, Changsha, China; 3 Chinese National Clinical Research Center On Mental Disorders (Xiangya), Mental Health Institute of the Second Xiangya Hospital, Central South University, Chinese National Technology Institute On Mental Disorders, Hunan Key Laboratory of Psychiatry and Mental Health, Changsha, China; 4 School of Humanities and Management, Hunan University of Chinese Medicine, Changsha, China; 5 Teaching and Research Section of Clinical Nursing, Xiangya Hospital of Central South University, Changsha, China

**Keywords:** Older adults, Anxiety, Depression, Risk factors, Community

## Abstract

**Background:**

Older adults’ psychological health is a public health issue that cannot be ignored, especially when these psychological health problems and related factors change across different social backgrounds because of rapid changes in traditions and family structures and the epidemic responses after the outbreak of COVID-19 in China. The aim of our study is to determine the prevalence of anxiety and depression and their associated factors among community-dwelling older adults in China.

**Methods:**

A cross-sectional study was conducted from March to May 2021 with 1173 participants aged 65 years or above from three communities in Hunan Province, China who were selected using convenience sampling. A structured questionnaire including sociodemographic characteristics, clinical characteristics, the Social Support Rating Scale (SSRS), the 7-Item Generalized Anxiety Disorder scale (GAD-7), and the Patient Health Questionnaire-9 Item (PHQ-9) was used to collect relevant demographic and clinical data and to measure social support status, anxiety symptoms, and depressive symptoms, respectively. Bivariate analyses were conducted to explore the difference in anxiety and depression based on samples’ different characteristics. The multivariable logistic regression analysis was performed to test for significant predictors of anxiety and depression.

**Results:**

The prevalence of anxiety and depression were 32.74% and 37.34%, respectively. Multivariable logistic regression analysis revealed that being female, being unemployed before retirement age, lacking physical activity, having physical pain, and having three or more comorbidities were significant predictors for anxiety. Subjective social support and support utilization were significant protective factors. Regarding depression, religion, lacking physical activity, having physical pain, having three or more comorbidities were found to be significant predictors. Support utilization was a significant protective factor.

**Conclusions:**

The study group showed a high prevalence of anxiety and depression. Gender, employment status, physical activity, physical pain, comorbidities, and social support were associated with psychological health problems of older adults. These findings suggest that governments should focus on the psychological health problems of older adults by raising community awareness of issues related to older adults’ psychological health. They should also screen for anxiety and depression among high-risk groups and encourage individuals to seek supportive counseling.

## Background

As life expectancies increase worldwide, older adult populations are aging faster than ever, which impacts almost all aspects of society. The global population of people aged 60 and above is expected to reach 1.4 billion by 2030 and 2.1 billion by 2050, and of this population, nearly 22% and 65% will live in low- and middle-income countries, respectively [1]. In the past decade, China has passed its first phase of rapid population aging and will soon face even more rapid population aging. In 2020, more than 190 million people in China were aged 65 or above, accounting for 13.50% of the total population. Further, the annual net increase in the older adult population is almost directly from the lowest in the twenty-first century to the highest (in 2023), and the older adult dependency rate has risen to 19.7% [2, 3]. Like most countries, China also faces significant challenges in their national economic, social security, and health systems when responding to this demographic shift, while older adults simultaneously face both physical and psychological health problems, that should also be given great emphasis [1, 2, 4].

As an integral and essential component of health, psychological health is as important in older age as it is at any other stage of life. Globally, over 20% of adults aged 60 and above have a mental or neurological disorder (excluding headache disorders). 6.6% of all disability among this age group is attributed to mental or neurological disorders, while depressive and anxiety disorders affect approximately 7% and 3.8% of this population, respectively [5]. Further, while a larger proportion of older adults experience anxiety and depressive symptoms that seriously affect their quality of life, they are unwilling to seek treatment or do not meet psychiatric diagnostic criteria [6]. Across cultures and regions, the prevalence of depression among older adults ranges between 11.4–36.7% [7–9], while the incidence of anxiety is between 14.2–39.4% [10–12]. In China, previous regional surveys have shown that the prevalence of anxiety and depression among older adults ranges between 11.77–22.3% and 26.5–40.3%, respectively [13–15]. Moreover, a cross-temporal meta-analysis indicated that the psychological health of older adults in China declines each year with the development of society [16]. Currently, Chinese policymakers and healthcare professionals have begun to focus on older adults’ psychological health; however, despite recent improvements, the nation still faces substantial challenges related to the workforce, healthcare expenditure, and service coverage [17, 18]. Older adults also often lack the ability to recognize and monitor problems in their own psychological health. In particular, the influence of traditional culture and national character can negatively impact their willingness to seek professional help and may even lead to feelings of shame [19].

Older adults’ psychological health has long been a concern in China. Previous research shows factors affecting the psychological health of older adults mainly included differences in socio-demographic details, physiological conditions, and social participation and social support [20–23]. However, many recent studies focus on the psychological status of older adults in the context of public health emergencies or within specific groups, including empty nesters and older adults with disabilities, chronic diseases, or other diseases [24–28]. Study outcomes have been broader and more diffuse, including quality of life, cognitive impairment, social support, and sleep quality [22, 23, 28, 29]. New reports of psychological health data for the general older adult population are still limited. Further, older adults’ psychological health and the related risk factors do not remain constant but instead change over time, especially in countries and regions with rapid social and economic development. Given the rapid changes in traditions and family structures and the regular epidemic responses after the outbreak of COVID-19 in China, we were interested in the psychological health problems of older adults at the current stage. However, previous studies on psychological health problems in older adults were mostly conducted before or during the outbreak of the COVID-19 pandemic, rather than regular epidemic prevention and control stage. Thus, it is necessary to identify the real status of psychological health more accurately among older adults at the current stage. The present study investigated the prevalence and risk factors of anxiety and depression among community-dwelling older adults in Hunan Province—as there were no studies investigating the situation in Hunan Province—to provide updated data and information on the psychological health of older adults. Up-to-date prevalence estimates are vital for service delivery, resource provision, and research developments. Likewise, identifying accurate prevalence variability and influence factors can help to address queries about etiology and advise the design of future studies.

## Methods

### Samples and procedure

**Fig. 1:**
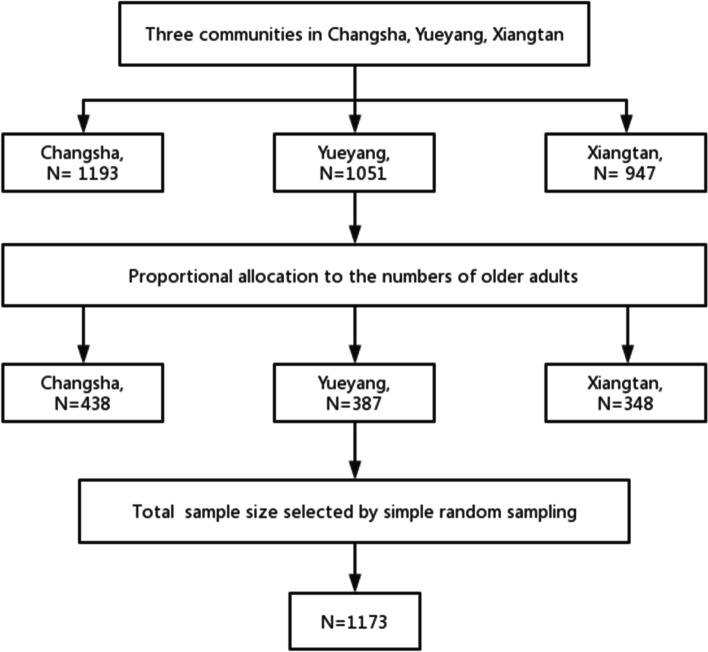
The sampling framework of this study

The study is a part of a research project that aims to assess the unmet psychological health needs of older adults in Hunan province, China. The data for this study were obtained from the field research of the subject team from Central South University in Changsha, Hunan Province. This cross-sectional study was conducted from March to May 2021 in Hunan Province, which is in China’s Central-southern region and has an area of 21.18 million square kilometers that accounts for 2.2% of China’s land area. According to national statistics reported in 2020, the total population of Hunan Province is 6.6 million, which accounts for 4.71% of the total national population [30]. Among the 34 provincial-level administrative regions in China, the old-age dependency ratio of Hunan Province ranks fourth highest. Hunan Province consists of 13 cities and one autonomous prefecture. According to the population proportion, Changsha, Yueyang, and Xiangtan were selected because they represent high, middle, and low levels of population density, respectively [31]. They are also representative areas characterized by high aging and the rapid development of psychological health for older adults. By the sample size calculation formula for a cross-sectional [32], $\mathcal{n}=\frac{{Z}_{{1-\alpha /2}^{2}}P(1-P)}{{\mathcal{d}}^{2}}$, at ${Z}_{1-\alpha }=$ 1.96, $\mathrm{\alpha }=$ 0.05, $\mathcal{d}=$ 0.05, and according to a previous study [33], the prevalence of depression in elderly was reported to be 30.6%. Considering a 20.0% non-response rate, at least 388 samples were required for the analysis. Inclusion criteria included: being aged 65 years or above, having lived in the surveyed communities for more than 6 months, being able to communicate with the investigators in a reasonable manner, and being sufficiently physically fit to respond the survey. Exclusion criteria included: being diagnosed with dementia or other serious mental health disorders, as reported by relatives. The sample of the study was drawn from three communities (one community in each of the three cities) by convenience sampling. The sample was proportionally allocated to each community based on the number of their older adults. A computer-generated simple random sampling method was employed to select older adults by using their sampling frame from each community. The community service center staff helped with participant recruitment using telephone methods, leading to the inclusion of 1173 individuals who met the inclusion criteria. The sampling framework of this study as shown in Fig. 1.

### Variables and instruments

In our study, psychological health problems encompass both anxiety and depression. Dependent variables included: anxiety (absent: GAD-7 scores of 0 ~ 4, present: GAD-7 scores of 5 ~ 21), depression (absent: PHQ-9 scores of 0 ~ 4, present: PHQ-9 scores of 5 ~ 27). Independent variables included: age (65–74y old, 75–89y old, ≧90y old), gender (female, male), BMI (underweight: < 18.5, normal: 18.5–23.9, overweight: 24–27.9, obese: ≧28), educational level (no formal education or primary education, secondary education, tertiary education), marital status (single, married, divorced, widowed), number of children (childless, one child, two children, three or more children), pre-retirement occupation (unemployed, mental labor, physical labor), monthly personal income (<1000￥($143.9); 1000–3000￥($143.9–$431.7); 3000–5000￥($431.7–$719.5); > 5000￥($719.5)), religion (no, yes), smoking (no, yes), physical activity level (no exercise, 1–2 times per week, ≧3 times per week, almost daily), physical pain rating (no physical pain, mild physical pain, moderate physical pain, severe physical pain), and comorbidities (no comorbidities, one comorbidity, two comorbidities, three or more comorbidities).

Trained investigators administered a pre-tested and structured questionnaire, which included sociodemographic characteristics, clinical characteristics, the Social Support Rating Scale (SSRS), the 7-Item Generalized Anxiety Disorder scale (GAD-7), and the Patient Health Questionnaire-9 Item (PHQ-9) in-person. The SSRS was used to measure the types and levels of social support older adults received. The SSRS was developed to reflect the Chinese context and comprised three dimensions: objective support, subjective support, and support utilization. The scale included 10 items consisting of three grades, with an aggregate score ranging from 7–56. Higher scores indicated higher levels of social support. Cronbach’s alpha for the SSRS was 0.92 for internal consistency [34]. The GAD-7 [35] was used to measure anxiety symptoms in older adults. It comprised seven items and each scored between 0–3 points, with total score ranging between 0–21. Scores of 5–9, 10–14, 15 and above represented mild, moderate, and severe anxiety symptoms, respectively. This scale was shown to have good internal and test–retest reliability, with a Cronbach’s alpha coefficient of 0.93 [36]. The PHQ-9 [37] was used to measure the severity of self-reported depressive symptoms in older adults. It includes nine items, each of which are scored between 0–3 scores, with total scores ranging between 0–27. Scores of 5–9, 10–14, 15 and above represent mild, moderate, and severe depressive symptoms, respectively. This scale was shown to have good internal and test–retest reliability, with a Cronbach’s alpha coefficient of 0.91 [38].

### Statistical analysis

Statistical analyses were performed using the Statistical Package for the Social Science (SPSS) version 20.0 for Windows. Descriptive statistics of participants’ sociodemographic and clinical characteristics were included as frequencies and proportion percentages for categorical variables, and mean values and standard deviations were included for continuous variables. Bivariate analyses using chi-squared tests were conducted to explore the differences between categorical variables groups. Correlations were determined to test linear relationships between variables. Multiple logistic regression analyses were performed using the enter method to estimate the associations between the study’s dependent (anxiety, depression) and independent variables (participants' sociodemographic details, clinical characteristics, and social support). Only variables found to be significant in the bivariate analyses were included in the multiple regression analyses. Adjusted odds ratios (AOR) and 95% confidence levels (CI) were used to quantify the strength of the association. Adjusted ORs were presented by controlling for other independent variables entered into the multiple logistic regression model. P-values less than or equal to 0.05 were considered statistically significant.

### Quality control and ethical considerations

Participant recruitment was performed in strict accordance with the inclusion and exclusion criteria, and the questionnaires were collected on the spot. The investigators received uniform training prior to administering the survey. During the data collection process, the investigators only explained and guided the questions, and were not allowed to induce or interfere with the participants’ responses. Two researchers performed data entry to ensure data accuracy, and if inconsistencies occurred, a third party would review the original data and make any necessary corrections. This study was reviewed and approved by the Medical Ethics Committee of Xiangya Hospital at Central South University (number 201909818). The study’s objectives, procedures, questionnaire content, and impact were explained to all enrolled respondents, and written informed consent was collected from each participant or their proxy prior to administering the questionnaire. Participants’ privacy and confidentiality were attentively ensured at every stage of the study. Those whose scores reflected severe anxiety or depressive symptoms were informed and advised to seek professional treatment at an available health clinic. We confirm that all methods were performed in accordance with the relevant guidelines and regulations (Declaration of Helsinki).

## Results

Sociodemographic and clinical characteristics showed that most of the participants (68.2%) were between the ages of 65–74 years, with an average age of 72.74 (SD: 6.47) years, and the oldest respondent was 101 years old. The proportions of female and male participants were 53.6% and 46.4%, respectively. Over 90% had a lower educational level, and 45.4% had not reached secondary education. Most of the respondents were married, had a pre-retirement occupation of physical labor or had been unemployed, had more than one child, and reported a monthly personal income below 3000 RMB ($431.7). Out of the sample, 23.9% of the respondents did not exercise, 31.6% exercised 1–2 times per week, 11.7% exercised three or more times per week, and 32.8% exercises almost daily. Regarding BMI, 56.7% were normal, 34.7% were overweight or obese. Nearly half of the respondents experienced varying degrees of physical pain, and the majority (79.4%) reported one and more comorbidities.

**Fig. 2:**
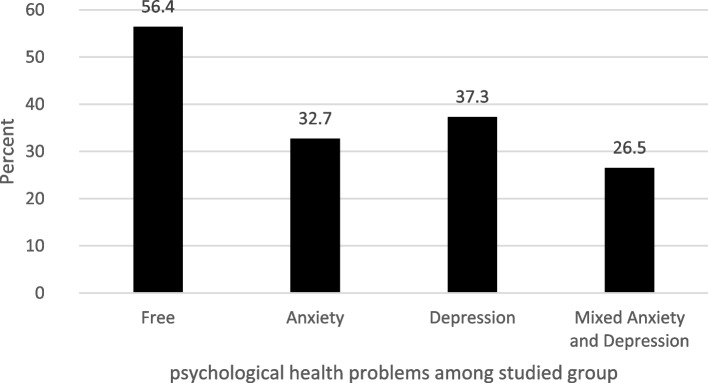
Psychological health problems among the studied group (*N* = 1173). Free, the group having neither anxiety nor depression; anxiety, the group having anxiety; depression, the group having depression; mixed anxiety and depression, the group having both anxiety and depression

**Fig. 3:**
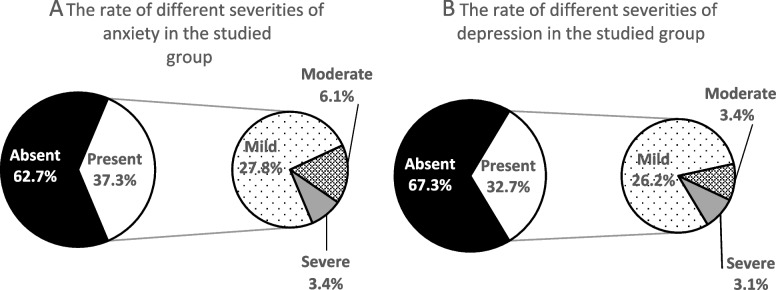
Rates of different severity levels of anxiety and depression (*N* = 1173). Absent_,_ GAD and PHQ scores of 0–4; present, GAD and PHQ scores of 5 and above; mild, GAD and PHQ scores of 5–9; moderate, GAD and PHQ scores of 10–14; severe, GAD and PHQ scores of 15 and above

**Fig. 4:**
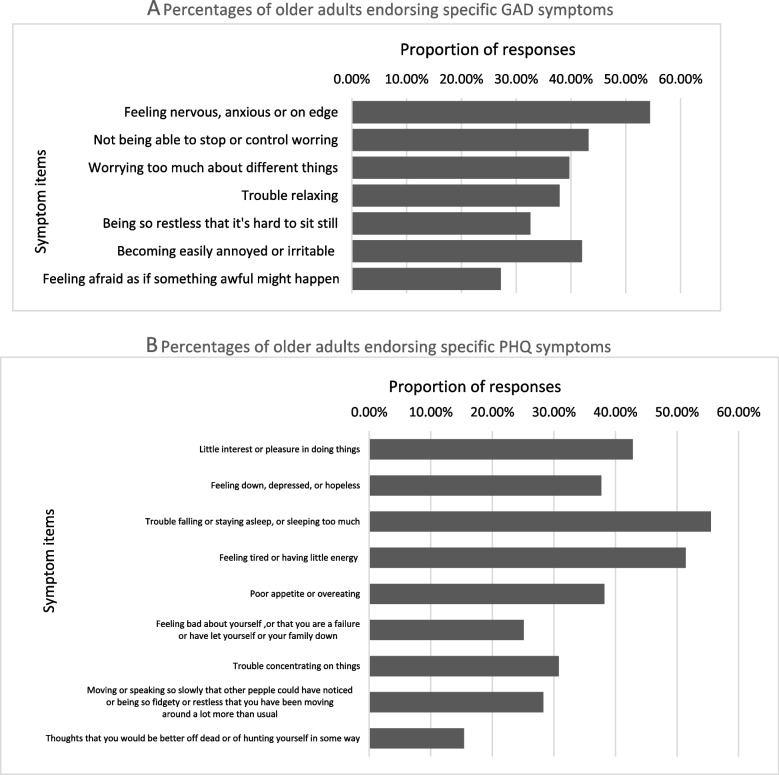
Percentages of older adults endorsing specific GAD and PHQ symptoms (*N* = 1173)

Figures 2 and 3 show the participants’ psychological health characteristics. In our study, participants were categorized based on whether psychological health symptoms were “absent” or “present.” For anxiety and depression, “absent” indicated that they had normal psychological health (GAD and PHQ scores of 0–4). Those in the “present” group were then subcategorized as “mild,” “moderate,” and “severe” (scores of 5–9, 10–14, or 15 and above, respectively). In total, 32.7% participants reported anxiety, 37.3% reported depression, and 26.51% reported mixed anxiety and depression. Further, as shown in Fig. 4, feeling nervous, anxious, or on edge (54.4%); not being able to stop or control worrying (43.2%); and becoming easily annoyed or irritable (42.0%) were the most common anxiety symptoms, while trouble falling or staying asleep or sleeping too much (55.5%); feeling tired or having little energy (51.4%); and little interest or pleasure in doing things (42.8%) were the most common depressive symptoms.

Table 1 shows the proportions of participants with anxiety and depression according to different sociodemographic and clinical characteristics, and the associations between those characteristics and psychological health problems. Females were more likely to have anxiety and depression compared to males (*P*_1_<0.001; *P*_2_ = 0.002); the older adults with low education levels were more likely to have anxiety and depression (*P*_1_ = 0.047;* P*_2_ = 0.003); different occupational status before retirement was associated with the incidence of anxiety and depression (*P*_1_<0.001; *P*_2_<0.001); older adults with low monthly personal income were more likely to have anxiety and depression compared to those with higher monthly personal income (*P*_1_ = 0.024; *P*_2_ = 0.007); less physical activity could also be associated with anxiety and depression (*P*_1_<0.001; *P*_2_<0.001). It was also worth noting that the older adults with relatively severe physical pain (*P*_1_<0.001; *P*_2_<0.001) and more comorbidities (*P*_1_ = 0.035; *P*_2_<0.001) were more likely to have anxiety and depression.

**Table 1:** Sociodemographic and clinical characteristics and related associations with psychological health problems (*N* = 1173)

Characteristics	Anxiety n(%)	Depression n(%)	*P*-value of x^2^-test	
**Age**				
65–74	274(34.3)	303(37.9)	*P*_1_ = 0.263	*P*_2_ = 0.857
75–89	104 (29.6)	127 (36.2)		
≧ 90	6 (27.3)	8 (36.4)		
**Gender**				
Female	238 (37.8)	260 (41.3)	*P*_1_<0.001	*P*_2_ = 0.002
Male	146 (26.8)	178 (32.7)		
**BMI**				
Underweight (< 18.5)	32 (31.7)	46 (45.5)	*P*_1_ = 0.125	*P*_2_ = 0.019
Normal (18.5–23.9)	214 (32.2)	237 (35.6)		
Overweight (24–26.9)	88 (30.6)	99 (34.4)		
Obese (≧ 27)	50 (42.4)	56 (47.5)		
**Educational level**				
No formal education or primary education	192 (36.0)	220 (41.3)	*P*_1_ = 0.047	*P*_2_ = 0.003
Secondary education	163 (31.0)	190 (36.1)		
Tertiary education	29 (25.4)	28 (24.6)		
**Marital status**				
Single	5 (33.3)	5 (33.3)	*P*_1_ = 0.379	*P*_2_ = 0.132
Married	283 (31.6)	319 (35.6)		
Divorced	71 (46.7)	6 (40.0)		
Widowed	89 (36.0)	108 (37.3)		
**Number of children**				
Childless	11 (39.3)	13 (46.4)	*P*_1_ = 0.228	*P*_2_ = 0.252
One child	65 (30.4)	76 (35.5)		
Two children	149 (30.5)	171 (35.0)		
Three or more children	159 (36.0)	178 (40.3)		
**Pre-retirement occupation**				
Unemployed	103 (42.9)	211 (35.7)	*P*_1_<0.001	*P*_2_<0.001
Mental labor	196 (33.2)	110 (32.2)		
Physical labor	85 (24.9)	117 (48.8)		
**Monthly personal income (1￥ is equal to about $0.1439)**				
< 1000￥($143.9)	108 (35.5)	131 (43.1)	*P*_1_ = 0.024	*P*_2_ = 0.007
1000–3000￥($143.9–$431.7)	170 (34.9)	178 (36.6)		
3000–5000￥($431.7–$719.5)	89 (30.7)	109 (37.6)		
> 5000￥($719.5)	17 (18.4)	20 (21.7)		
**Religion**				
No	354 (31.9)	401 (36.2)	*P*_1_ = 0.018	*P*_2_ = 0.001
Yes	30 (46.2)	37 (56.9)		
**Smoking status**				
No	290 (31.9)	326 (35.9)	*P*_1_ = 0.281	*P*_2_ = 0.060
Yes	94 (35.5)	112 (42.3)		
**Physical activity level**				
No exercise	118 (42.1)	132 (47.1)	*P*_1_<0.001	*P*_2_<0.001
1–2 times per week	138 (37.2)	164 (44.2)		
≧ 3 times per week	43 (31.4)	45 (32.8)		
Almost daily	85 (22.1)	97 (25.2)		
**Pain rating**				
No physical pain	163 (24.7)	183 (27.8)	*P*_1_<0.001	*P*_2_<0.001
Mild physical pain	10 (26.3)	16 (42.1)		
Moderate physical pain	121 (43.5)	136 (48.9)		
Severe physical pain	90 (45.5)	103 (52.0)		
**Comorbidities**				
No comorbidities	85 (33.6)	86 (34.0)	*P*_1_ = 0.035	*P*_2_<0.001
One comorbidity	118 (29.3)	130 (32.3)		
Two comorbidities	91 (31.0)	108 (36.7)		
Three or more comorbidities	90 (40.4)	114 (51.1)		

*P*_*1*,_ *P*-value for the differences in prevalence of anxiety among subjects with different characteristics; *P*_*2*,_ *P*-value for the differences in prevalence of depression among subjects with different characteristics

Table 2 shows the correlations between psychological health problems and the study’s continuous variables, including age and social support. Anxiety was negatively correlated with age, subjective support, support utilization, and SSRS total score, while depression was negatively correlated with SSRS total score and each dimension of the scale.

**Table 2:** Correlations among participants’ age, social support, and psychological health problems (*N* = 1173)

Variables	Anxiety		Depression	
	*P*-value	**Correlation coefficient**	*P*-value	**Correlation coefficient**
Age	0.003	-0.088	0.447	-0.022
Subjective support	0.001	-0.101	<0.01	-0.139
Objective support	0.303	-0.3	0.011	-0.074
Support utilization	<0.01	-0.172	<0.01	-0.182
SSRS total score	<0.01	-0.119	<0.01	-0.159

Table 3 and Table 4 show the results of the multivariable logistic regression analysis conducted to test for significant predictors of anxiety and depression. Based on the results of the bivariate analysis, significant variables were accessed in the regression model. In the multivariable model, those of female gender showed a higher risk factor for anxiety (AOR 1.448; 95%CI 1.100–1.906). Being unemployed before retirement age was a risk factor for anxiety (AOR 1.865; 95%CI 1.188–2.928). Physical activity at a level less than 1–2 times per week was a risk factor for anxiety and depression (AOR_1_ 1.946; 95%CI_1_ 1.387–2.730; AOR_2_ 2.316; 95%CI_2_ 1.661–3.229). In addition, moderate physical pain or greater severity (AOR_1_ 2.226; 95%CI_1_ 1.619–3.061; AOR_2_ 2.371; 95%CI_2_ 1.725–3.259) and having three or more comorbidities (AOR_1_ 1.430; 95%CI_1_ 1.020–2.003; AOR_2_ 1.830; 95%CI_2_ 1.313–2.550) were also significantly associated with an increased risk of anxiety and depression. For social support, we found support utilization to be a protective factor for anxiety and depression (AOR_1_ 0.836; 95%CI_1_ 0.786–0.910; AOR_2_ 0.869; 95%CI_2_ 0.815–0.926).

**Table 3:** Significant predictors for anxiety in the respondents (*N* = 1173)

Characteristics	Anxiety (*N* = 1173)			
	UOR	UOR 95%CI	AOR	AOR 95%CI
**Age**	0.980	(0.962, 1.000)	0.980	(0.958, 1.002)
**Gender**				
Female	1.659***	(1.294, 2.128)	1.448**	(1.100, 1.906)
Male	1^a^		1^a^	
**Educational level**				
No formal education or primary education	1.650*	(1.045, 2.607)	1.137	(0.826, 1.565)
Secondary education	1.316	(0.831, 2.085)	1.196	(0.666, 2.146)
Tertiary education	1^a^		1^a^	
**Pre-retirement occupation**				
Unemployed	1.500**	(1.112, 2.023)	1.865**	(1.188, 2.928)
Physical labor	2.273***	(1.595, 3.239)	1.238	(0.877, 1.876)
Mental labor	1^a^		1^a^	
**Monthly personal income (1￥ is equal to about $0.1439)**				
< 1000￥($143.9)	2.431**	(1.366, 4.327)	1.176	(0.836, 1.653)
1000–3000￥($143.9–$431.7)	2.366**	(1.353, 4.136)	1.207	(0.791, 1.841)
3000–5000￥($431.7–$719.5)	1.953*	(1.091, 3.498)	0.699	(0.345, 1.417)
> 5000￥($719.5)	1^a^		1^a^	
**Physical activity level**				
No exercise	2.571***	(1.833, 3.605)	2.213***	(1.533, 3.195)
1–2 times per week	2.090***	(1.518, 2.878)	1.946**	(1.387, 2.730)
≧3 times per week	1.615**	(1.046, 2.491)	1.532	(0.972, 2.416)
Almost daily	1^a^		1^a^	
**Pain rating**				
Mild physical pain	1.087	(0.517, 2.286)	1.001	(0.457, 2.194)
Moderate physical pain	2.345***	(1.745, 3.153)	2.226***	(1.619, 3.061)
Severe physical pain	2.536***	(1.821, 3.531)	2.326***	(1.635, 3.310)
No physical pain	1^a^		1^a^	
**Comorbidities**				
Three or more comorbidities	1.51**	(1.118, 2.040)	1.430**	(1.020, 2.003)
Less than three comorbidities	1^a^		1^a^	
**Support utilization**	0.880***	(0.833, 0.929)	0.836***	(0.786, 0.910)
**Subjective support**	0.963**	(0.940, 0.985)	0.930**	(0.869, 0.995)
**Total social support score**	0.975**	(0.961, 0.990)	1.053	(0.993, 1.105)

1^a^, Reference category; UOR, Unadjusted Odds Ratio; AOR, Adjusted Odds Ratio (adjusted for all variables); *p < 0.05, **p < 0.01, ***p < 0 .001

**Table 4:** Significant predictors for depression in the respondents (N = 1173)

Characteristics	Depression (N = 1173)			
	UOR	UOR 95%CI	AOR	AOR 95%CI
**Gender**				
Female	1.449**	(1.140, 1.840)	1.223	(0.933, 1.602)
Male	1^a^		1^a^	
**BMI**	1.043	(0.896, 1.214)	1.038	(0.881, 1.223)
**Educational level**				
No formal education or primary education	2.159**	(1.363, 3.420)	1.075	(0.786, 1.471)
Secondary education	1.737**	(1.094, 2.757)	0.664	(0.369, 1.194)
Tertiary education	1^a^		1^a^	
**Pre-retirement occupation**				
Unemployed	2.006***	(1.428, 2.818)	0.883	(0.610, 1.277)
Physical labor	1.171	(0.083, 1.553)	1.515	(0.975, 2.354)
Mental labor	1^a^		1^a^	
**Monthly personal income (1￥ is equal to about $0.1439)**				
< 1000￥($143.9)	2.726***	(1.581, 4.701)	0.924	(0.660, 1.294)
1000–3000￥($143.9–$431.7)	2.074**	(1.222, 3.518)	1.124	(0.745, 1.695)
3000–5000￥($431.7–$719.5)	2.168**	(1.251, 3.756)	0.635	(0.320, 1.259)
> 5000￥($719.5)			1^a^	
**Religion**				
Yes	2.330**	(1.405, 3.864)	2.089**	(1.200, 3.637)
No	1^a^		1^a^	
**Physical activity level**				
No exercise	2.648***	(1.906, 3.678)	2.181***	(1.515, 3.139)
1–2 times per week	2.352***	(1.729, 3.201)	2.316***	(1.661, 3.229)
≧3 times per week	1.452	(0.950, 2.220)	1.377	(0.876, 2.164)
Almost daily	1^a^		1^a^	
**Pain rating**				
Mild physical pain	1.892	(0.972, 3.683)	1.769	(0.859, 3.642)
Moderate physical pain	2.491***	(1.863, 3.331)	2.371***	(1.725, 3.259)
Severe physical pain	2.820***	(2.034, 3.910)	2.452***	(1.722, 3.491)
No physical pain	1^a^		1^a^	
**Comorbidities**				
Three or more comorbidities	2.021***	(1.505, 2.714)	1.830***	(1.313, 2.550)
Less than three comorbidities	1^a^		1^a^	
**Subjective support**	0.951***	(0.929, 0.973)	0.972	(0.943, 1.002)
**Objective support**	0.978	(0.943, 1.015)	1.047	(0.998, 1.098)
**Support utilization**	0.867***	(0.822, 0.914)	0.869***	(0.815, 0.926)

1^a^, Reference category; UOR, Unadjusted Odds Ratio; AOR, Adjusted Odds Ratio (adjusted for all variables); *p < 0.05; **p < 0.01; ***p < 0 .001

## Discussion

In the present study, the prevalence of anxiety and depression among older adults were found to be 32.7% and 37.3%, respectively. These figures are in line with some previous studies, including Ma J et al. [22], who reported respective rates of 34.2% and 38.9% among 1587 community-dwelling older adults in China. Cho et al. [12] reported respective rates of 39.4% and 35.6% among 655 community-dwelling older adults in Myanmar. However, other studies report different results. Chuang et al. [39] reported rates of 43.6% and 39.7% for anxiety and depression, respectively, among 204 older adults in rural areas of Beijing. Abdul et al. [40] also reported lower prevalence rates of 27.8% and 22.6%, respectively, for 230 rural community-dwelling older adults in Malay, while Xie Q and Gang Tian [21, 26] reported respective rates of 21.1% and 26.75% in older adults in China. It appears difficult to compare with rates of anxiety and depression reported in other studies because of differences in respondents and national cultures. In various regions of Europe and Africa, the prevalence of anxiety and depression among older adults were 14.1%–20.8% (significantly lower) and 32.2%–47.1% (significantly higher), respectively [10, 41]. For our study, our research data were collected throughout the regular epidemic responses stage after the outbreak of COVID-19 in China. Although the study participants were all in low-risk areas, the public health policies adopted included special area regional blockades and closures of some social gathering places. Because of this, older adults faced possible declines in various functional aspects of life and increased difficulties in conducting their daily activities [42], which might have affected their psychological health to varying degrees. According to previous research, the highest rates of anxiety and depression during the COVID-19 outbreak were 49.7% and 47.2%, respectively, with the prevalence of depression in this population increasing significantly from 7.2% to 19.8% since the beginning of the pandemic [43, 44].

The presence of anxiety was found to be significantly related to gender, with female participants more likely to experience anxiety symptoms. However, in the multivariate analysis, no association was found between gender and depressive symptoms. These findings are partly consistent with those of previous studies. For example, a systematic review found that gender was the sociodemographic factor most frequently associated with anxiety and depression in older adults, and this relationship was supported by several studies [44, 45]. Ahmed et al. [11] found gender to be a significant predictor for anxiety and depression and reported a higher prevalence of anxiety among women compared with men (16.0% and 10.7%, respectively). Work, economic, educational, neuro-hormonal, psychological, and genetic aspects may mediate this gender disparity [44–47]. In the context of Chinese traditional culture, differences in education were present in that generation, leading older adult women to be more likely to have a lower educational level compared with their male counterparts and less likely to be previously employed, which can lead to financial difficulties [20]. Older adult women also tend to have fewer hobbies and lack spiritual sustenance, which can result in poor social support [15]. In the general family structure, older adult women often care for their grandchildren and undertake a heavier burden within the family division of labor, which can lead to interpersonal disputes and family conflicts [8]. However, this phenomenon has been reversed in New China, and men and women now have equal opportunities for education and a more balanced family division of labor. Differences can also be explored from a biological angle. From a genetic perspective, men and women have significant dimorphic gene expression patterns in key areas of the brain, and gender differences in the immune system, neuroplasticity, and certain hormone and neurotransmitter markers have been known for decades [7]. Therefore, a combination of the above factors can result in low self-expectations and negative attributional patterns and higher negative affect among women.

In this study, most participants (68.2%) were between the ages of 65–74 years, and the average age was 72.74 (± 6.47) years. Age was not entered into the multivariate logistic regression as a predictor of anxiety or depression. This finding is in line with Behera et al. [7] and Zhao et al. [48], but inconsistent with Robb et al. [49], who found that older age is a protective factor for depression or anxiety, with every five-year increase in age resulting in a 19% (OR 0.81; 95%CI 0.77–0.85) and 22% (OR 0.78; 95%CI 0.75–0.83) lower risk of reporting worsening symptoms of depression and anxiety, respectively. A study of Chinese older adults found that anxiety levels in people aged 60–71 years were significantly higher than in those aged 72–92 years. This indicates that geriatric anxiety can differ between age groups [50]. Consequently, a necessary factor to consider in the present study is that the comparisons between ages when calculating ORs were made within a relatively similar range. This may have led to less significant risk compared to age groups with greater differences, as a risk factor for anxiety and depression may exist at a particular age. In addition, because of increased age, older adults face increased risk of physical disease, which manifested as an increase in age, creating the illusion of an increased incidence of anxiety and depression, rather than it being a direct effect of age [44].

Associations between pre-retirement occupation and anxiety or depression were reported in this study, aside from the results presented in Table 3. When using the unemployed elderly population as a reference group, in terms of anxiety, both mental (OR 0.543; 95%CI 0.345–0.853) and physical (OR 0.688; 95%CI 0.494–0.958) labor were protective factors, while physical labor was a protective factor for depression (OR 0.581; 95%CI 0.416–0.812). These findings are in line with Park et al. [51], who found that being employed (either mental or physical labor) could lead to older adults feeling less anxious and depressed because they are able to contribute to their family and community. This work also created opportunities of social participation and social roles for the dependent. Further, specific occupations might affect psychological health, as demonstrated by a study conducted in Spain that reported that being a civil servant and being retired are protective factors for depression [52]. In general, older adults with some types of employment might belong to a higher social class and have a higher income; thus, they may be highly valued and treated with respect. Hence, as the occupational status of older adults can help account for differences in their quality of life and psychological health status, additional jobs should be created for older adults to improve their quality of life and mental health [53].

In this study, active participation in physical exercise was found to be a significant protective factor for anxiety and depression. Previous studies also reported similar findings. For example, Songheun et al. [54] compared an exercise and a non-exercise group, finding that exercise can affect depressive symptoms among older adults. Carlos et al. [55] reported that regular exercise may represent a protective factor for anxiety and depression, while Park et al. [56] found that an exercise-based program had positive effects on promoting subjective health status, improving life satisfaction, and relieving depression among older adults. Proper physical activity directly relieves negative emotions, such as stress, and is also associated with better physical fitness and increased motor function, especially in older populations [44]. Good motor function helps older adults improve their ability to live their daily lives, meet their basic physiological needs, also satisfy their psychological needs, such as those for social contact and entertainment [57]. Additionally, cognitive function, anxiety, and depression in older adults have been found to be correlated, and older adults who exercise regularly generally have better cognitive function than those who do not exercise [58]. Therefore, older adults should regularly participate in various exercise and physical activity programs to improve their mental health by improving their cognitive and motor function.

In total, 78.5% of this study’s participants reported having at least one comorbidity, and 44.1% reported having two or more. The presence of comorbidities and the number of diseases were significantly associated with both anxiety and depression in this study, and older adults with three or more comorbidities were found to be more likely to develop psychological health problems. This is in line with previous studies [12, 14, 59], which found that unhealthy older adults were more likely to experience anxiety and depression, while older adults without chronic illnesses generally had better overall mental health. Liu [60] noted that when older adults experience complications related to chronic diseases, their probability of depression will increase nearly twofold with each increase in the number of chronic diseases. For older adults with comorbidities, physical function could be impaired and their ability to perform activities of daily living could also be limited, which may lead to a decline in quality of life [61]. Moreover, chronic illnesses themselves can create stigma for those who experience them, leading them to become more self-critical than healthy older adults [62]. Based on the above, comorbidities must be addressed through effective healthcare systems so that older adults can reduce their risk of psychological challenges.

In this study, high social support utilization was found to be a protective factor for anxiety and depression in older adults, indicating the important role social support plays in improving psychological health among this population. Previous literature supports the moderating effect of social support on older adults’ psychological health, showing that older adults with better social support are less likely to have symptoms of anxiety or depression, and poor social support may be an important factor for psychological problems [49, 63]. Notably, in the present study, objective social support and total social support did not appear to be significance for psychological health among older adults. These results have a few possible explanations. First, all participants in the current study were community-dwelling, and most of them lived in urban areas with relatively good economic conditions; thus, they have adapted to living in groups and reside in areas with good infrastructure [64]. However, although they have good social security, such as medical care and pensions, they also have higher material and spiritual requirements; when those are unsatisfied, it is easy to experience negative emotions [65]. Second, according to a previous local survey conducted in China [66], only 40% of older adults have received medical services provided by the community, indicating that the community has not yet fully utilized its advantages of convenient medical services to cater to older adults, and the medical and health service system still has a long way to go. Finally, objective support mainly includes material support and direct services, and compared with material support, emotional support (e.g., the company of their grandchildren, telephone calls with their children) has a greater effect on promoting older adults’ psychological health [67]. This indicates that higher objective support does not directly lead to better psychological health for older adults, and subjective support and support utilization may be more important. Hence, older adults need to be able to accept care and help from others to obtain positive support and strengthen their utilization of social support [29]. Support institutions could promote older adults’ psychological health by advocating for the younger generation to provide emotional support and strengthening communication among spouses, children, and older adults to reduce loneliness and the sense of loss and enhance older adults’ ability to cope with stressful situations, thereby reducing their risk of psychological problems [13, 15].

This study’s results also revealed that physical pain is a risk factor for anxiety and depression, which is consistent with Cabak’s [68] previous study, in which psychological health was markedly poorer in patients with chronic pain compared with healthy controls, and among participants who occasionally or regularly consumed analgesics compared with those who did not. First, possible explanations for this finding include that physical pain directly causes unpleasant experiences for older adults. Second, the consequences of chronic pain may also be important factors, including restriction of physical activity, high disability rates, and increased anxiety and depression when worrying about the side effects of analgesic drugs [23]. Third, negative emotions also affect how individuals perceive pain, which may lead them to develop negative beliefs and enhance cues for pain sensation, thereby forming a vicious cycle between psychological health problems and physical pain [69].

Finally, in the present study, a lack of religious affiliation was a protective factor for depression, compared with religious older adults, which is contrary to the findings of previous studies [63]. The reason for this may be that, in China, most citizens have no religious tradition, and some individuals only develop religious beliefs when they experience a major life change to cope with traumatic events and ease their negative emotions. In other words, because many people may have depression before they become religious, people without depression are even less likely to experience religion. Thus, no religious affiliation might be a protective factor in the study. However, this was a limitation of our study: the cross-sectional design limited the understanding of the causality between the factors and outcomes. While religion has a positive significance, it also has limitations. A study has found that religious believers were at higher risk for post-traumatic stress disorder (PTSD) when faced with major trauma [70]. At times, religion may be an additional burden, which makes the individual’s psychological problems more serious [71].

Our study revealed a high prevalence of psychological health problems among community-dwelling older adults in the current social background. Female gender, pre-retirement unemployment, lack of physical activity, physical pain, having three or more comorbidities, less social support and support utilization were found to be significant predictors of psychological health problems. These findings indicate the demand for psychological healthcare services for this population. Relevant departments should continue to work on the system of policies to protect older adults’ rights and interests, improve social pensions and medical services, and build a psychological support system for older adults by collaborating with individuals, families, and the community. Community-level medical institutions could be an alternative channel for early detection of psychological health problems, if the staff were trained to screen for psychological health problems like anxiety and depression among older adults with comorbidities and other high-risk groups. Moreover, community institutions could also organize activities and provide regular exercise programs for older adults, to strengthen bonds among older adults, increasing their social support, improve their support utilization, and encourage them to seek supportive counseling.

This study has several limitations. First, convenience sampling was used in this study, and the participants were limited to urban community residents in Hunan. This limits the generalization of our findings to other studies. Second, the data were self-reported and dependent on the respondents’ subjective assessments, may also have led to recall bias. No objective measures were incorporated including the cognitive capacity of older adults, therefore, the indicated levels of anxiety and depression may not always be consistent with the evaluations of psychological health professionals. Third, the study was cross-sectional, so although we examined several pieces of sociodemographic information and clinical characteristics related to older adults’ psychological health problems, no cause-effect relationship can thus be established. Fourth, not all factors were included in the modeling, including older adults’ social interactions and living arrangements. In subsequent studies, all factors need to be included in the modeling for a more robust understanding.

Despite these limitations, based on the background of regular epidemic prevention and control after the outbreak of COVID-19, our study is a new one that represents a significant step in understanding psychological health conditions among older adults in China. The results add to the existing literature on updated information on the prevalence and associated factors of anxiety and depression in older adults, which are vital for service delivery, resource provision, and research developments.

## Conclusions

Now, given the rapid changes in traditions and family structures, and the regular epidemic responses after the outbreak of COVID-19 in China, our study revealed information about psychological health status and related factors among community-dwelling older adults in Hunan Province, China. There is a high prevalence of anxiety and depression among the study group. Being of female gender, being unemployed before retirement age, lacking physical activity, having physical pain, having three or more comorbidities, and having less social support and support utilization were found to be significant predictors of psychological health problems. These results indicate that it is critical to identify psychological health problems more effectively among this population. Given the different effects of the sociodemographic information, coping strategies should also be reflective of the demographic disparities in this vulnerable population. Further support might be provided accordingly to improve the psychological health of older adults.

## Data Availability

The datasets used and/or analyzed during the current study are available from the corresponding author on reasonable request.
